# Genetic diversity of Murray Valley encephalitis virus 1951–2020 identified via phylogenetic and evolutionary analyses

**DOI:** 10.1371/journal.pntd.0013181

**Published:** 2025-07-03

**Authors:** Timo Ernst, Alice Michie, Chisha Sikazwe, Jay Nicholson, Avram Levy, I.-Ly Joanna Chua, John S. Mackenzie, David W. Smith, Allison Imrie

**Affiliations:** 1 School of Biomedical Sciences, University of Western Australia, Perth, Western Australia, Australia; 2 PathWest Laboratory Medicine WA, Perth, Western Australia, Australia; 3 Environmental Health Directorate, Public and Aboriginal Health Division, Department of Health, Perth, Western Australia, Australia; 4 Faculty of Health Sciences, Curtin University, Perth, Western Australia, Australia; 5 Medical School, University of Western Australia, Perth, Western Australia, Australia; Oregon State University College of Veterinary Medicine, UNITED STATES OF AMERICA

## Abstract

Murray Valley encephalitis virus (MVEV) is a mosquito-borne orthoflavivirus endemic to Australia that can cause fatal neurological disease. The enzootic focus of MVEV is believed to reside in northern Western Australia (WA). We sequenced whole genomes of 70 MVEV sampled over 51 years, 1969–2020, from locations across Australia and Papua New Guinea (PNG) and identified greater MVEV diversity than previously recognized. Genotype 1 (G1) demonstrated greatest intra-genotype diversity and was predominant over the sampling period with sub-lineage G1B circulating in WA and seeding activity across Australia. G1A included viruses sampled across northern WA, as well as the Northern Territory (NT). A newly identified sub-lineage G1C circulated in northern WA in 1993 and was detected again in 2003. G2 viruses were distributed across the Kimberley and Pilbara regions of northern WA, and in the NT. Although no new G3 and G4 viruses, previously identified only in PNG, were detected in the present study, other MVEV originating in PNG clustered with G1A. We confirm MVEV is enzootic in northern WA, with transmission occurring more frequently and across a wider geographical area than previously recognised. Additionally, we identify evidence of regular genotype replacement that has occurred over many decades where the major genotypes G1 and G2 have circulated in northern WA since the late 1960s. We also show that WA MVEV likely seeded an MVE outbreak in Victoria in 1974, further supporting the notion that the enzootic focus of MVEV lies in northern WA. Recent increases in MVEV detections, MVE cases and deaths in WA and across Australia highlight the need for enhanced surveillance and more frequent sampling to understand viral origin and genomic diversity, to identify potential virulence motifs, and to understand the ecological drivers that determine emergence of MVEV in northern WA and movement of MVEV across the country.

## Author summary

We analysed MVEV sequenced from archived virus isolates and mosquito homogenates collected since 1972 by the Western Australia Arbovirus Surveillance Program and by various research projects, with other published whole and partial genome sequences, to understand MVEV genetic diversity, evolution, and geographic distribution over a 69-year period 1951–2020. We amplified MVEV from virus isolates and developed an MVEV tiling amplicon PCR assay to analyse mosquito homogenates, and sequenced whole genomes of 70 viruses to create the largest and most geographically and temporally diverse MVEV dataset published to date. We describe a new sub-lineage G1C that circulated unrecognised in the 1990s, highlighting the value of more regular and strategic virus sampling and genome sequencing to capture the true diversity and evolutionary history of MVEV.

## Introduction

Murray Valley encephalitis is a mosquito-borne disease caused by infection with Murray Valley encephalitis virus (MVEV), a zoonotic orthoflavivirus [1] that belongs to the Japanese encephalitis virus (JEV) serogroup along with other medically important arboviruses including West Nile virus (WNV), WNV (Kunjin subtype) (KUNV), Usutu virus (USUV) and St Louis encephalitis virus (SLEV) [2].

MVEV was first isolated in 1951 in Australia from post-mortem brains of fatal cases during an outbreak in the Murray Valley and surrounding regions [3,4]. Prior to introduction of Japanese encephalitis virus to Australia in 2021 [5,6], MVEV infection has historically caused the most severe disease of any of the endemic arboviruses of Australia. While most MVEV infections are asymptomatic or cause a nonspecific febrile illness, an estimated 1 in 800 cases develop meningitis or encephalitis among whom the case fatality rates are greater than 20% [7,8]. Long-term neurological complications including flaccid paralysis occur in 30–50% of survivors. As the asymptomatic and mild cases are rarely diagnosed, the epidemiology of MVEV remains poorly understood.

Humans are considered dead-end hosts of MVEV. Water birds, such as herons and egrets, act as reservoir hosts and amplify virus in a cycle that exists between birds and mosquitoes [9,10]. Other animals including kangaroos, rabbits and horses may be incidentally infected [11,12]. The main mosquito vector in Australia is *Culex annulirostris*, which breeds in freshwater habitats, and the virus may also be vectored by other *Culex* species and some *Aedes* species [13]. *Cx. annulirostris* is present throughout Australia and is a vector for several medically important orthoflaviviruses and alphaviruses.

MVEV activity is monitored in Australia with mosquito-based genomic surveillance and sentinel chicken serosurveillance programs conducted regularly by most states [14–16]. MVEV is enzootic in northern Australia [17–22], with regular activity detected in the remote Kimberley region of Western Australia (WA) and in the Top End region of the Northern Territory (NT). Human cases occur most frequently in the Kimberley region and in NT. Activity is epizootic in the Pilbara and Gascoyne regions south of the Kimberley, and in central Australia, and also occasionally in south-eastern Australia particularly during times of heavy rain and flooding of the Murray-Darling River system. Virus transmission is thought to be facilitated by movement of viremic waterbirds into newly flooded regions that were previously arid, and by persistence of virus in desiccation-resistant mosquito eggs [23]. However, these patterns of virus activity may be modified by environmental manipulations [24]. Outside Australia, MVEV has been isolated in Papua New Guinea (PNG) [25–27] and a case of Murray Valley encephalitis in the Papua province of Indonesia in 1960 was diagnosed using serological tests [28].

Four genotypes (G1-G4) of MVEV have been identified [29,30] based on complete and partial genomes of viruses sampled in Australia and PNG. G1 is the dominant group in mainland Australia and includes the prototype MVEV strain MVE-1–51, isolated from the brain of a fatal human case of encephalitis during an outbreak of MVE in Victoria (VIC) in 1951 [4]. Two G1 sub-lineages have been previously described [31] based on analysis of prM/E gene sequences. Historically, G1A has been largely restricted to WA, while G1B viruses are found in WA and have circulated in the NT and across the eastern and southern Australian states other than Tasmania. The prototype G2 strain OR156 was isolated from *Cx. annulirostris* mosquitoes trapped in Kununurra in northern WA in 1973 [32], and G2 viruses have so far been mostly identified in northern WA with one isolate originating in the NT [33]. The prototype G3 strain NG156 was isolated from the brain of a fatal human encephalitis case in Port Moresby, PNG in 1956 [25]. This strain is sometimes referenced as MVE-1–56 and is the only known G3 isolate. Similarly, strain MK6684, isolated from a pool of Culicine mosquitoes collected in PNG in 1966 at Japanaut on the Sepik River in East Sepik Province [26], is the only known representative of G4.

Currently there are only nine MVEV whole genomes publicly available, with five belonging to G1, two to G2 and one each for G3 and G4 [12,30,33–36]. In this study we derived complete or near complete viral genomes from an archival collection maintained at UWA and PathWest Laboratory Medicine WA and analysed these sequence data with the few known complete MVEV genomes and with published partial genome sequences.

## Materials and methods

### Viruses

Genome sequencing was conducted on 70 MVEV isolates or mosquito homogenates sampled in PNG and various locations across Australia from 1969 – 2020 (S1 Table and S1 Fig). Each pool contained 1–25 mosquitoes of the same species. MVEV was previously identified in mosquito homogenates by PCR or by fixed cell ELISA conducted on infected cell monolayers [37,38]. WA MVEV samples were sourced from an archival virus collection obtained through the ongoing, state-wide arbovirus surveillance program that began in 1987, and from other surveillance and research projects conducted since the early 1970s. The majority of isolates were derived from mosquito specimens, most commonly from *Cx. annulirostris*. An historical MVEV isolate T69 was isolated from an individual thought to have been infected near the border between WA and the NT in 1969 and who subsequently died in Brisbane, Queensland.

### Virus amplification and RNA extraction

A subset of viruses (n = 34) was sequenced using an approach we have described previously [39]. Briefly, frozen archival virus isolates were passaged once on Vero (African green monkey kidney cells; ATCC: CCL-81) or C6/36 (*Aedes albopictus* larval cells; ATCC: CRL-1660) cell monolayers. Viral RNA was then extracted from clarified, concentrated supernatant with the Roche High Pure RNA extraction kit [39]. Extracted RNA was quantitated using the Qubit RNA High Sensitivity (HS) assay kit (Thermofisher) prior to library preparation using the TruSeq Stranded mRNA library preparation kit (Illumina), as per standard protocol without poly-A tail selection steps [39].

### Complementary DNA synthesis and tiling amplicon RT-PCR

The remaining viruses (n = 36) were sequenced with a tiling amplicon approach, involving generation of overlapping PCR amplicons that span the entire MVEV genome. Viruses sequenced in this manner were not amplified by cell culture, but instead were directly sequenced from RNA extracted from archival mosquito homogenates or low passage viral stocks stored at -80°C, using the Roche High Pure RNA extraction kit as per standard protocol.

Complimentary DNA was synthesized from extracted RNA using SuperScript IV VILO Master Mix (Thermofisher). The cDNA reaction mix contained 3 μL of Superscript IV VILO Reaction Mix, 9 μL of nuclease free water, and 3 μL of extracted RNA template. The reaction mix was incubated at 25°C for 10 minutes, 50°C for 25 minutes and 85°C for 5 minutes. The synthesized cDNA was used as input in tiling PCR reactions.

Two separate primer schemes were designed using Primal Scheme software [40], with specificity for the G1 and G2 MVEV genotypes, producing roughly 1200 bp and 2000 bp amplicons, respectively (S2 Table and S2 Fig). Both assays could be multiplexed, by separately pooling in equal volume the odd and even numbered primers from 10 μM stock.

The PCR reaction mixture contained 4.5 μL viral cDNA, 12.5 μL Platinum SuperFi II Green PCR Master Mix, 7 μL nuclease free water and 1 μL of combined forward and reverse primer (10μM stock). The PCR incubation conditions involved an initial denaturation at 98°C for 30 seconds followed by 35 cycles of 98°C for 10 seconds, 60°C for 10 seconds and 72°C for 30 seconds. A final extension was performed for a single cycle at 72°C for 5 minutes. All PCR products were individually visualized with a Gel Red (Biotium)-stained agarose gel. PCR amplicons from the same sample were pooled based on the intensity of the gel product, prior to purification with AMPure XP beads. The purified pools were diluted to 0.2 ng/μL as input for library preparation using the Nextera XT library preparation kit (Illumina), as per standard protocol.

### Sequencing

Libraries prepared with either the TruSeq or the Nextera XT library preparation kits were sequenced separately on a MiSeq instrument, with standard v2 (2 x 150 bp) flow cells. Reads were trimmed of adapters and PhiX reads, before undergoing *de novo* genome assembly within Geneious (2021.2.2) or CLC Genomics Workbench (v.8.5.1).

### Phylogenetic analysis

Two datasets were created. One contained 79 complete or near complete MVEV CDS whilst the second was comprised of 131 sequences of 2,004 bp encompassing the prM and E genes and included previously published and newly sequenced isolates. Each dataset was aligned using MAFFT v7.450 [41] as implemented in Geneious Prime (2021.2.2). The CDS alignment was examined for evidence of recombination using RDP4 [42] with the following algorithms (with default conditions): RDP [43], BootScan [44], MaxChi [45], Chimaera [46], 3seq [47], GENECONV [48], LARD [49] and SiScan [50]. Both alignments were assessed using jModelTest v2.1.10 [51] and a GTR + Γ_4_ + Ι model was determined to be most suitable for both according to the Akaike information criterion. Phylogenetic reconstruction was carried out using RAxML v8.2.11 [52] with 1,000 bootstrap replicates and visualized in FigTree v1.4.4. Both phylogenies were rooted with the corresponding JEV sequence (GenBank accession number NC_001437).

### Temporal analysis

Both datasets were analyzed for temporal signal through a root-to-tip regression analysis using TempEst v1.5.3 [53]. There was poor temporal information in the CDS dataset (with an R^2^ value of 0.044) and thus only the prM/E gene dataset (R^2^ = 0.35) was examined using Bayesian analysis. A Bayesian Markov Chain Monte Carlo (MCMC) method as implemented in BEAST v1.10.4 [54] was used to estimate the divergence times from the most recent common ancestor (MRCA) as well as evolutionary rates. The analysis of the prM/E gene dataset used a relaxed molecular clock model (uncorrelated lognormal), with a general time reversible nucleotide substitution model with partitions at each codon position (1 + 2 + 3) and a Bayesian skyline coalescent tree prior. The MCMC chains were run for 100 million generations, with convergence being assessed using Tracer v1.7.1 [55]. For all parameters, the observed effective sample sizes were greater than 200. To visualise the results, a maximum clade credibility (MCC) phylogeny was constructed using TreeAnnotator (available as part of the BEAST package), with a 10% burn-in.

### Genotype and sub-lineage distribution and abundance

A third dataset was generated, comprised of the sequences included in the prM/E gene dataset as well as all additional MVEV E gene sequences available on GenBank (S3 Table). This dataset contained 246 sequences, and was further refined to remove any duplicates leaving 200 unique complete or partial E gene sequences. An alignment of these 200 E gene sequences was generated using MAFFT v7.450 [41] as implemented in Geneious Prime (2021.2.2) and a RAxML phylogeny reconstructed [52] with 1000 bootstrap replicates. The phylogeny was visualised in FigTree v1.4.4 and all newly added sequences were assigned a genotype and sub-lineage, where possible. The geographical distribution and relative abundance of G1 and its sub-lineages, and G2, were visualised by generating heatmaps as implemented in QGIS v3.14, using a 150 km radius.

### Selection pressure detection

Selection pressure analyses were performed on the CDS dataset, using the DataMonkey server ( [56] https://www.datamonkey.org/) to expand the investigations undertaken by Williams *et al.* (2015) [31] based on a smaller prM/E gene dataset. Single likelihood ancestor counting (SLAC) and fixed effect likelihood (FEL) methods were used to identify sites for diversifying selection [57]. Episodic and pervasive diversifying selection were tested for using the mixed effects model of evolution (MEME) and fast unbiased Bayesian approximation (FUBAR) methods respectively [58,59].

Detailed protocols are available on request.

## Results

### Phylogenetic analysis

A total of 70 complete or near complete MVEV coding sequences were generated in this study, expanding the available whole MVEV genomes from nine to 79. No evidence of recombination was detected in the CDS alignment using RDP4 with the eight implemented algorithms.

**Fig 1:**
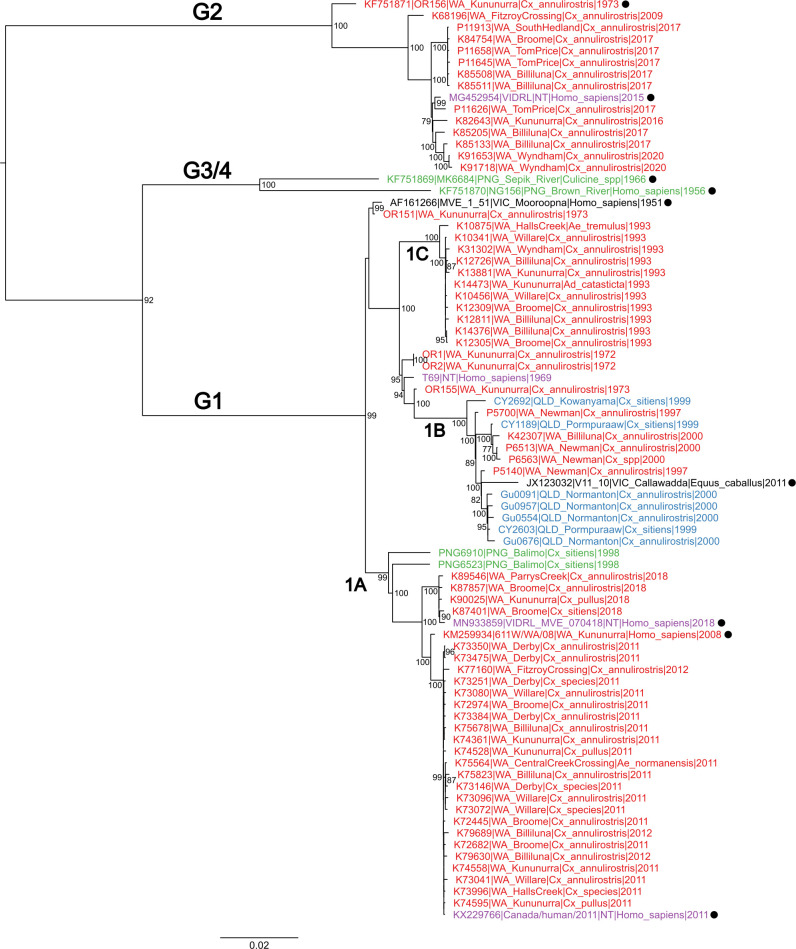
Maximum likelihood phylogenetic tree of 79 MVEV complete and near-complete polypeptide coding sequences. Genotypes and subtypes are indicated on the phylogeny, whilst the geographic origins are indicated by colour: Northern Territory (NT), purple; Queensland (QLD), blue; Victoria (VIC), black; Western Australia (WA), red; Papua New Guinea (PNG), green. Previously published sequences are indicated by ●. The phylogeny was estimated using a general time-reversible model with a gamma distribution (4 categories) and invariant sites. Bootstrap support of nodes is indicated for 1000 replicates, with values ≥75% displayed. The phylogeny was rooted using a JEV (GenBank accession number: NC_001437) CDS that was removed to improve visual fidelity.

Phylogenetic analysis was conducted on the CDS (Fig 1) and prM/E gene (S3 Fig) datasets using maximum likelihood methods. Both datasets produced phylogenies with identical topologies, with the four known genotypes of MVEV clearly identifiable. In each phylogeny, G2 viruses were basal to the remaining MVEV genotypes. The distinction between the clade containing G3 and G4, and the G1 clade, is well supported in the CDS dataset (bootstrap of 92%) but was poorly supported in the prM/E dataset (bootstrap of 69%). The previously identified sub-lineages of G1 (G1A and G1B) [31] were well supported in both phylogenies.

G1 was the predominant MVEV genotype sampled in the present study with 57 of 70 viruses (81.4%) belonging to this group. G1 is comprised of samples with diverse geographic origins spanning most Australian states and territories (NSW, NT, QLD, VIC and WA) as well as PNG. G1A viruses were predominantly sampled from northern WA over the period 2008–2018 and clustered into three major clades apparent in CDS and prM/E phylogenies, covering three time periods: 2008–2009, 2011–2012 and 2018. Two other viruses, sampled from a symptomatic person from the NT in 2018 and from a traveller infected in the NT and who died of MVE on their return to Canada in 2011, also belonged to G1A as did two viruses from PNG sampled in 1998 [29,34,36]. Viruses identified as belonging to sub-lineage G1B originated from WA, NT, NSW, QLD, NSW and VIC. G1B viruses could be divided into two clades, based on the prM/E gene phylogeny, one of which spanned 1997–2002 (WA and QLD), and the other that spanned 2002–2011 (WA, QLD, NSW and VIC) (S3 Fig).

In this present study we have identified a clade of viruses sampled in northern WA which we have designated G1C, that is distinct from other G1 viruses. G1C viruses circulated during 1993–1994 within the Kimberley, and were sampled again in 2003. The G1C clade was isolated predominantly from *Culex* species mosquitoes, with some evidence of vectoring by *Aedes* species, specifically *Aedes tremulus* and *Aedes normanensis*. Interestingly, the phylogeny indicates that this cluster of viruses persisted within the Kimberley, as evidenced by a virus from this genotype being sampled again a decade later in 2003. There have been no further detections of G1C since 2003 and this clade may now be extinct, although it is also possible GIC may circulate undetected in remote areas of WA where surveillance is not regularly conducted.

G2 comprised sequences sampled from the Kimberley and Pilbara regions of WA between 1973 and 2020, and included a virus sampled outside WA, in the NT in 2015 [33]. All WA isolates were sampled from trapped mosquitoes whereas the NT virus was isolated from a patient with encephalitis [33]. Of the 70 sequences generated in the present study, 13 (18.6%) belonged to G2.

No new G3 or G4 viruses were identified. To date, both G3 and G4 each consist of a single virus – the G3 isolate that was derived from a fatal encephalitis case in 1956 [25] and the G4 virus isolated from mosquitoes trapped in 1966 [26], both from PNG. In the present study we identified two other viruses originating in PNG, PNG6523 and PNG6910 isolated from mosquitoes trapped in 1998, both clustered within G1A [31], highlighting the viral diversity that likely exists in PNG.

Intra- and inter-genotype pairwise distances were calculated for the CDS and for each individual gene region (S4 Table). G1 nucleotide identities ranged from a minimum of 91.9% (NS2B) to a maximum of 100% (all genes), whereas the minimum intra-genotype identity for G2 viruses was determined to be 95.6% (NS2B). A consistent finding across the genome was that intra-genotype diversity was greatest in G1 viruses whereas the inter-genotype diversity was greatest for G2 viruses. This trend was previously identified in analyses of the prM and E genes and is consistent with the phylogenetic analyses [31].

### Evolutionary history

**Fig 2:**
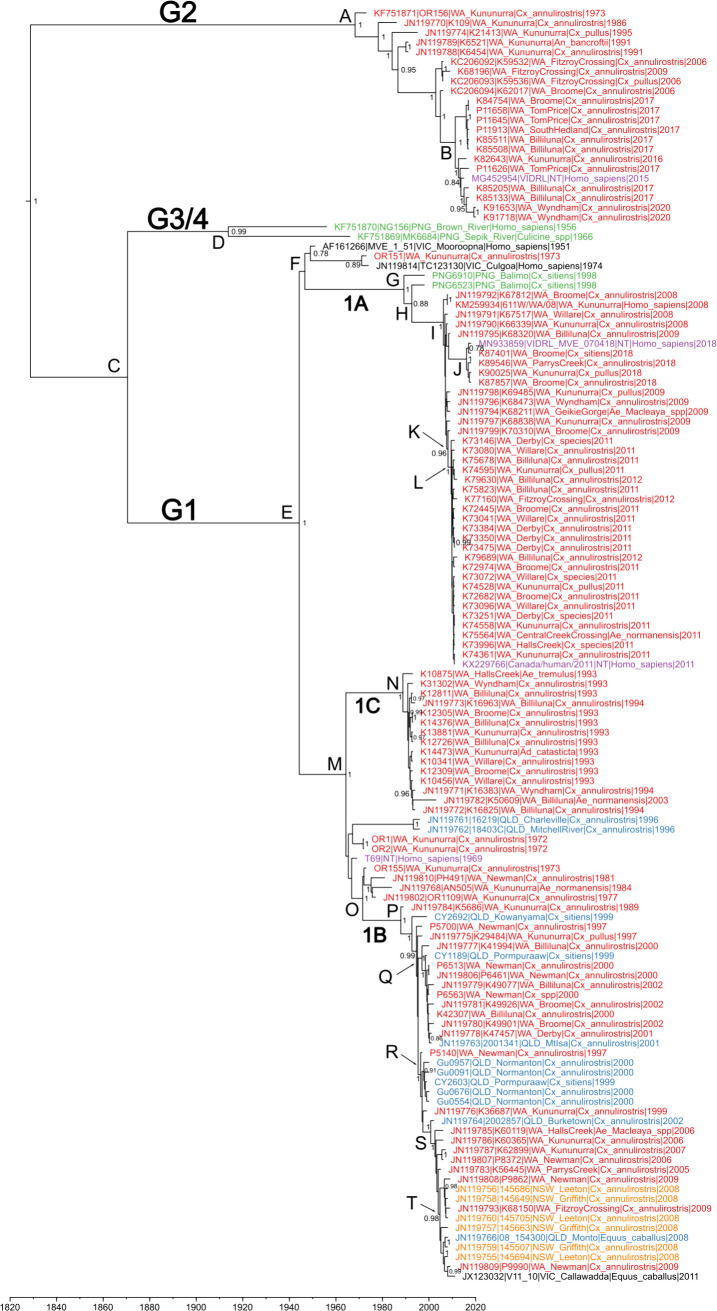
Maximum clade credibility tree of 131 MVEV prM/E gene sequences. Genotypes and subtypes are indicated on the phylogeny, whilst the geographic origins are indicated by colour: New South Wales (NSW), orange; Northern Territory (NT), purple; Queensland (QLD), blue; Victoria (VIC), black; Western Australia (WA), red; Papua New Guinea (PNG), green. Posterior support probability is shown for nodes with values ≥ 0.75. Letters adjacent to nodes indicate divergence times and nucleotide substitution rates shown in Table 1.

Root-to-tip regression analyses were performed on both the CDS and prM/E gene datasets. Clock-like signal was observed for the prM/E gene dataset (R^2^ = 0.35), but not in the CDS dataset (R^2^ = 0.04). Only the prM/E gene dataset, therefore, was analyzed through a Bayesian MCMC analysis to estimate the MRCA and evolutionary rates of genotypes and clades of interest (Fig 2). An estimated MRCA of 1829, with a 95% highest probability density (HPD) between 1712 and 1902 was determined for all MVEV sequences (Table 1). Genotype 2 viruses emerged from this ancestral MVEV and continued to evolve, whilst the ancestor of genotypes 1, 3 and 4 diverged in 1870 (node C). Genotypes 3 and 4 diverged in approximately 1914 (node D), whilst the estimated MRCA for G1 isolates was 1944 (node E).

**Table 1:** Estimated divergence dates and mean substitution rates for MVEV genotypes and selected clades from the Bayesian analysis.

Node	Major clade	Divergence date		Mean substitution rate (subs/site/year)	
		Year	95% HPD	Rate	95% HPD
Root	All	1828.74	1712.12-1902.19	7.16 x 10^–4^	5.15 x 10^–4^ to 9.29 x 10^–4^
A	G2	1968.25	1957.37-1973	1.11 x 10^–3^	1.59 x 10^–4^ to 2.33 x 10^–3^
B	–	2011.27	2009.37-2013.01	8.39 x 10^–4^	3.62 x 10^–4^ to 1.38 x 10^–3^
C	G1, G3, G4	1870.48	1804.82-1920.48	7.90 x 10^–4^	1.08 x 10^–4^ to 2.03 x 10^–3^
D	G3, G4	1913.76	1869.5-1942.59	7.07 x 10^–4^	1.32 x 10^–4^ to 1.56 x 10^–3^
E	G1	1944.26	1934.8-1950.36	1.01 x 10^–3^	1.52 x 10^–4^ to 2.10 x 10^–3^
F	–	1946.63	1940.1-1950.79	6.69 x 10^–4^	6.95 x 10^–5^ to 1.53 x 10^–3^
G	G1A	1989.23	1977.3-1995.07	2.98 x 10^–4^	9.91 x 10^–5^ to 6.61 x 10^–4^
H	–	1992.6	1983.88-1996.52	6.65 x 10^–4^	9.63 x 10^–5^ to 1.55 x 10^–3^
I	–	2006.2	2003.67-2007.46	8.72 x 10^–4^	3.88 x 10^–4^ to 1.38 x 10^–3^
J	–	2016.12	2014.17-2017.24	7.06 x 10^–4^	2.69 x 10^–4^ to 1.18 x 10^–3^
K	–	2008.18	2007.44-2008.91	7.25 x 10^–4^	1.19 x 10^–4^ to 1.63 x 10^–3^
L	–	2009.51	2008.77-2010.24	7.41 x 10^–4^	1.55 x 10^–4^ to 1.54 x 10^–3^
M	–	1964.28	1957.87-1967.96	4.51 x 10^–4^	9.96 x 10^–5^ to 9.54 x 10^–4^
N	G1C	1988.77	1984.64-1991.44	6.37 x 10^–4^	2.48 x 10^–4^ to 1.19 x 10^–3^
O	–	1971.51	1969.12-1972.94	8.29 x 10^–4^	2.41 x 10^–4^ to 1.64 x 10^–3^
P	G1B	1987.87	1984.65-1989	8.72 x 10^–4^	4.76 x 10^–4^ to 1.21 x 10^–3^
Q	–	1996.99	1995.29-1998.29	9.00 x 10^–4^	1.92 x 10^–4^ to 1.91 x 10^–3^
R	–	1997.76	1996.61-1998.73	7.07 x 10^–4^	1.14 x 10^–4^ to 1.57 x 10^–3^
S	–	2000.71	1999.24-2001.84	1.36 x 10^–4^	5.11 x 10^–4^ to 2.44 x 10^–3^
T	–	2004.72	2003.17-2006.16	6.36 x 10^–4^	1.17 x 10^–4^ to 1.41 x 10^–3^

The MRCA of G1A was estimated to be 1989 (95% HPD 1977–1995). Excluding two PNG viruses sampled in 1998, the majority of G1A is comprised of viruses sampled from WA between 2008–2018. These viruses were sampled in three distinct time-periods (2008–2009, 2011–2012 and 2018). The MRCA of these viruses are indicated by node I, with an estimated emergence date of 2006. The MRCA of clades from 2011-2012 and 2018 are indicated by nodes L and J respectively (Table 1). Interestingly, the 2018 sub-clade is not descendant from the 2011–2012 clade, but instead, from viruses sampled between 2008–2009.

G1B was estimated to have emerged slightly earlier than G1A in approximately 1988 (95% HPD 1985–1989). This sub-lineage contained the most geographically diverse members of all MVEV genotypes/clades, although most viruses, overall, were sampled in WA. MVEV that were sampled during an MVE outbreak in 2000 in which seven and nine cases were diagnosed in the NT and WA respectively [60], formed a sub-clade within G1B with an MRCA of 1997 (node Q). A small cluster of sequences from QLD sampled between 1999–2000 was observed with an MRCA of 1998 (node R) and MVEV sampled from WA, NSW, QLD & VIC between 2008–2011 had an MRCA of 2005 (node T).

Viruses sampled in WA during an oubtreak that occurred between 1993–1994 formed an intermediary G1 clade, G1C, that did not cluster with the previously identified G1A or G1B sub-lineages. An MRCA of 1989 (95% HPD 1985–1991; node N) was estimated with this clade last sharing a common ancestor with other G1 viruses in 1964 (node M). The evolutionary rate for G1C was calculated at 6.37 x 10^-4^ substitutions per site per year (HPD: 2.48 x 10^-4^ to 1.19 x 10^-3^), a rate that is consistent with other contemporary G1 genotypes, indicating that the genetic evolution within G1C proceeded at a similar pace as other genotypes.

G2 viruses are not well represented in the dataset possibly due to a lower frequency of circulation compared to G1 viruses. The last common ancestor of all identified G2 viruses was estimated to have been circulating in 1968 (95% HPD 1957–1973, node A). One clade was sampled between 2015–2020, with a MRCA estimated to be 2011 (95% HPD 2009–2013, node B). The mutation rate for this more recent clade was 8.39 x 10^-4^ substitutions per site per year (HPD: 3.62 x 10^-4^ to 1.38 x 10^-3^), slightly lower than the overall G2 mutation rate. This clade encompasses all MVEV sampled from WA in 2016, 2017 and 2020 as well as one virus sampled in NT in 2015.

The mean nucleotide substitution rate for all MVEV prM/E gene sequences was determined to be 7.16 x 10^-4^ substitutions per site per year (95% HPD: 5.15 - 9.29 x 10^-4^). There was minimal variation in the estimated evolutionary rates of individual clades (ranging from 2.98 - 11.1 x 10^-4^ subs/site/year), with all demonstrating overlapping 95% HPD intervals.

### Genotype and sub-lineage distribution and abundance

**Fig 3:**
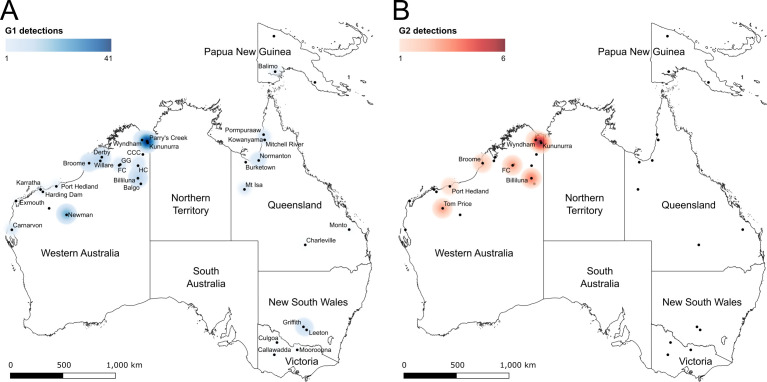
Geospatial heatmaps of MVEV genotypes 1 and 2 distribution across Australia and Papua New Guinea. The distribution of 163 G1 (panel A) or 21 G2 (panel B) viruses across Australia and PNG. Sites where MVEV were detected are indicated as points; place names are included to indicate when genotype designation was possible. Abbreviated place names are as follows: CCC – Central Creek Crossing, FC – Fitzroy Crossing, GG – Geikie Gorge, HC – Halls Creek. Heatmap radius was set to 150 km. The map was created by combining the shapefile of Australia provided by the Australian Bureau of Statistics (available at https://www.abs.gov.au/statistics/standards/australian-statistical-geography-standard-asgs-edition-3/jul2021-jun2026/access-and-downloads/digital-boundary-files/STE_2021_AUST_SHP_GDA2020.zip) with a map of Papua New Guinea (available at https://geojson-maps.kyd.au/) using QGIS v3.14.

**Fig 4:**
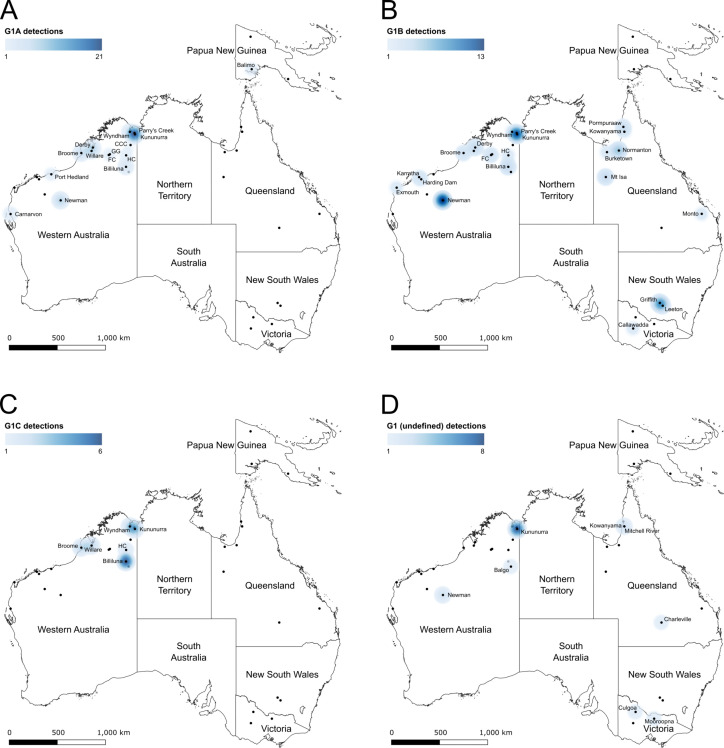
Geospatial heatmaps of MVEV genotype 1 sub-lineages across Australia and Papua New Guinea. The distribution of G1A (panel **A)**, G1B (panel **B)**, G1C (panel C) or G1 sub-lineage undefined (panel **D)**, viruses across Australia and PNG. Sites where virus lineages were detected are indicated through place names. Abbreviated place names are as follows: CCC – Central Creek Crossing, FC – Fitzroy Crossing, GG – Geikie Gorge, HC – Halls Creek. Heatmap radius was set to 150 km. The map was created by combining the shapefile of Australia provided by the Australian Bureau of Statistics (available at https://www.abs.gov.au/statistics/standards/australian-statistical-geography-standard-asgs-edition-3/jul2021-jun2026/access-and-downloads/digital-boundary-files/STE_2021_AUST_SHP_GDA2020.zip) with a map of Papua New Guinea (available at https://geojson-maps.kyd.au/) using QGIS v3.14.

A total of 69 additional MVEV E gene sequences were combined with the prM/E gene dataset, with the phylogenetic analysis identifying all additional sequences as G1 viruses (S4 Fig). Of these 69, 39 were identified as belonging to G1A, 25 belonged to G1B, one belonged to G1C and a further four did not cluster with any of the identified sub-lineages (S3 Table). Geospatial heatmaps were then generated for G1 (Fig 3A) and G2 (Fig 3B) viruses as well as the sub-lineages of G1 (Fig 4).

G1 viruses were identified across the breadth of northern Australia, with three detected in the NT (although specific geospatial data are missing [34,36]) (S3 Table), and down eastern Australia into VIC. All G1 viruses were sampled between 1951 and 2018, with the foci of detections occurring in the vicinity of Kununurra in northern WA. Sub-lineage G1A viruses were predominantly sampled in the vicinity of Kununurra, with detections occurring across the northern region of WA as well as in the NT and PNG between 1998 and 2018. G1B viruses were identified across northern WA, QLD, NSW and VIC between 1989 and 2011. G1C viruses circulated in the the Kimberley region of northern WA and were predominantly sampled in 1993 and 1994, with one virus detected in 2003. Interestingly, the foci of sub-lineage G1C was located in the vicinity of Billiluna, approximately 483 kilometres (300 miles) south of Kununurra.

G2 viruses were more geographically restricted, being detected in the Kimberley and Pilbara regions of northern WA with an additional detection in the NT [33]. All G2 viruses were sampled between 1973 and 2020, and similar to G1 viruses, the foci of detections occurred in the vicinity of Kununurra.

G1 viruses that did not cluster with one of the three identified sub-lineages G1A, G1B or G1C were detected across Australia between 1951 and 1996, with a foci located around Kununurra.

### Selection pressure analysis

Analyses for selection pressure were performed to identify if any amino acid sites across the MVEV genome were under selective pressure during the evolutionary history of the virus. A dN/dS ratio of 0.049 was estimated from the analyses implemented on the DataMonkey server, indicative of predominantly negative (purifying) selection. Three sites were identified as being under positive (diversifying) selection (S5 Table). One site, in the Envelope protein (S332G) was identified in three of the four methods used. The Env S332G substitution, located in the B-C loop of domain III that is a key region for interactions between virus and host cell [61,62] was identified predominantly in G1A sampled in 2011–2012, as well as in three isolates of G1B (sampled in 2000) and one G1C isolate sampled in 1993.

The remaining two sites were both identified in the NS4B protein, E24G & D107G, by one (MEME) & two (FUBAR & MEME) methods, respectively. The E24G substitution was found almost exclusively in the G1A 2011–12 clade, and in a single G1B isolate sampled in 1997 (P5140). The D107G substitution was identified across all genotypes, in 34 (47.2%) sequences. G1C isolate K10456 sampled in 1993 and G1B isolates K42307, P6513 and P6563 sampled in 2000, whilst possessing the E protein S332G substitution, did not possess the E24G substitution in the NS4B protein. This suggests that while the E S332G substitution might contribute to certain aspects of viral evolution or vector-host interaction, it alone does not appear to drive significant changes in transmission dynamics.

## Discussion

We have conducted the most comprehensive genome-scale phylogenetic analysis of MVEV to date, significantly expanding upon the existing genome-scale MVEV phylogeny that is based on nine genomes. We sequenced 70 complete or near complete MVEV genomes from virus isolates and mosquito homogenates collected over 51 years between 1969–2020 across WA, the NT, QLD and PNG. Additionally, we assessed the temporal history of MVEV sampled 1951 – 2020 using Bayesian evolutionary and selection analyses.

Over 75% of MVEV included in this investigation were sampled within WA, largely from mosquitoes collected in the remote Kimberley region in the north of the state at the end of the wet season, typically between March and April. The Kimberley is a sparsely populated area of over 420,000 square kilometres (over 160,000 square miles) where towns and communities are separated by great distances. Surveillance to monitor mosquito-borne disease is generally targeted around these populated areas and this relatively limited sampling likely means viral diversity is underestimated. WA-derived MVEV was sourced from archived tissue culture virus isolates and mosquito homogenates sampled in research projects and surveys since the 1970s, or collected for the WA Arbovirus Surveillance program since it began in 1987. MVEV is detected in most years. Limited sequence data from other Australian states and from PNG reflects more restricted surveillance with fewer samples available for analysis.

The estimated MRCA of 1828.74 (95% HPD: 1712.12-1902.19) for all sampled MVEV is consistent with that identified previously [31]. A mean substitution rate of 7.16 x 10^-4^ is within the bounds of what has previously been estimated from a smaller dataset of prM/E gene sequences and consistent with rates estimated for other members of the JE serocomplex [63–66].

G1 was the predominant MVEV genotype, with most viruses belonging to this group, and was comprised of samples with diverse geographic origins spanning most Australian states and territories (WA, NT, QLD, NSW and VIC) as well as PNG. A small cluster of viruses in G1 collected in the period 1969–1973 included samples from the east Kimberley and one virus from a fatal case who died in QLD, in Brisbane, and who may have acquired the virus while camping in the east Kimberley region of WA, close to the NT border. It is of note that a virus from the same period, MVEV OR151 that was sampled in 1973 from the east Kimberley clustered with the human-derived virus TC123130 that originated on the east coast of Australia, isolated in VIC during the major Australia-wide 1974 MVE outbreak, suggesting this outbreak in southeastern Australia was seeded from northern WA.

Detection of G1A in northern WA and the NT in 2018, descendant from viruses sampled in 2008–2009 in WA in the same region, indicates that there was unsampled diversity of the 2008–2009 clade continuing to circulate in northern WA in 2018 that was not identified during the intervening 2011–2012 outbreak.

There were three MVE outbreaks in WA during the current study period, in 1993, 2000 and 2011. All MVEV sampled from mosquitoes during these outbreaks belonged to G1 and clustered within distinct monophyletic clades. Viruses sampled during the 1993 outbreak formed a new, distinct monophyletic clade we have named G1C. All viruses sampled in 2000 grouped within sub-lineage G1B while 2011 viruses grouped within G1A. In each of the three WA outbreaks the sequenced viruses were relatively homogenous, reflecting rapid movement of viruses within WA across distances of up to 650 km (400 mi), most likely by waterbirds. In WA the 2000 G1B outbreak was characterized by an extreme southerly spread with MVE cases detected in regions with more temperate climate, distinct from the tropical/subtropical climate in the enzootic areas, and well outside the regions where disease typically occurs [19,67]. This 2000 outbreak was associated with highest-on-record rainfall across much of the state [19].

Each of these three WA outbreaks was characterised by high levels of MVEV activity in mosquito-based surveillance with associated high number of MVEV cases and deaths. The 1993 G1C outbreak resulted in nine cases (with one death) in WA and a further six cases (with one death) in the NT [60]. The 2000 WA G1B outbreak extended to the NT where seven cases were reported, with one death [60]. In 2011 there was widespread MVEV activity across broad swathes of Australia [68,69], with seventeen cases of MVE nationally. The majority of cases were identified in WA (9 cases, one death) and NT (four cases, one death), during a very active monsoon season with heavy rainfall. Additional cases were reported from South Australia (SA) (two cases, one death) and NSW (two cases) [22,34]. MVEV was detected in mosquitoes and humans and was also identified in a major outbreak of encephalitis in horses, mainly in NSW and VIC [70]. All MVEV sequences from WA and the NT in 2011 belonged to sub-lineage G1A. Interestingly, the single publicly available whole-genome sequence from VIC in 2011 originated from the outbreak among horses and clustered with sub-lineage G1B [12]. These parallel, widespread MVEV outbreaks in 2011 caused by two different sub-lineages and which occurred in the northwest and the southeast of Australia in the same time period suggest that these outbreaks were likely driven more by differing ecological factors, such as extreme weather conditions brought on by La Nina events, rather than virological factors. Further research is needed to understand the drivers of MVEV emergence across Australia.

A notable finding of this study has been the widespread detection of G2 in both the Kimberley and Pilbara regions of WA in 2016, 2017 and 2020. Previously, G2 had only been detected in the Kimberley region [29,31]. Additionally, one sequence in this clade was sampled in the NT in 2015, from a child with encephalitis [33]. This case from the NT is the first recorded instance of a G2 virus being recovered from a patient diagnosed with MVE and the first from outside WA, and indicates this group of viruses circulates outside northern WA. The G2 clade has been infrequently detected compared to G1, with far fewer virus isolates available for analysis. All recent G2 viruses shared a MRCA of 2011.27 (95% HPD 2009.37-2013.01), indicating that the progenitor of this clade was likely circulating during the significant 2011 G1A outbreak in WA. The limited number of previous G2 detections and the apparently confined geographical distribution within northern WA had led to the postulate that these viruses occupied a narrow ecological niche with restricted spread via mosquitoes and/or water birds [29,31]. Our current finding that G2 circulated over great distances within WA, and in the neighbouring NT, highlights the importance of more regular sampling across larger geographical areas to increase the likelihood of detecting virus and to better capture the true diversity of MVEV.

Interestingly, the G1 and G2 lineages have persisted since they were first isolated in the late 1960s (G1) and early 1970s (G2) although they were not always sampled in the same year. We observed over a decade-long period (2010–2020) a pattern whereby there was apparent cycling of dominating genotype sampled over a vast geographic span, in quick succession. For example, G1 circulated 2011–2012 and was replaced by G2 in 2015–2017, followed by G1 in 2018–2019 and replaced again by G2 in 2020. Viruses were sampled from mosquitoes that have been trapped at the same locations across the vast areas of northern WA, over the many decades of the surveillance program. This pattern of apparent genotype replacement may suggest frequent introduction and/or dissemination of MVEV from various locations within northern WA, NT and QLD, from PNG, or from other countries in the region where MVEV has not been described. We have previously described whole genome phylogenies for the endemic alphaviruses Ross River virus, Barmah Forest virus and the Sindbis-like Argyle virus over their decades of circulation in WA and did not observe this same pattern of long term genotype persistence and replacement and instead, identified fixed replacement of dominant genotypes by newly emergent variants [39,71,72]. Additional sampling to expand the number of MVEV sequences from NT, QLD, southeast Australia, PNG and potentially the Asia Pacific region, would allow tracking of MVEV genetic changes to assess evolutionary origin and virus movement, and identify emergence of new variants.

This is the first time that the CDS of a large MVEV dataset has been examined for evidence of recombination. Although recombination has been detected in other orthoflaviviruses including members of the JEV serocomplex, notably JEV [73–76], through computational and/or experimental methods none was detected in the present analysis.

Our finding of predominantly negative, purifying selection across the MVEV CDS is consistent with findings for other viruses of the JE serocomplex and orthoflaviviruses in general. The three sites identified as being under positive, diversifying selection at E S332G, NS4B E24G and NS4B D107G were present in a subset of viruses across all G1 lineages. The locations of these sites, in the B-C loop of E domain III that is a key region for virus-host interactions [61,62], and the the N-terminal region (1–125 aa) of orthoflavivirus NS4B that inhibits the antiviral innate immune response by blocking IFN-alpha/beta signalling [77], highlight possible roles in virus persistence, transmission, or pathogenicity. The NS4B proteins, encoded by different orthoflaviviruses and sharing the same topology, are important for viral replication [78] and play a pivotal role in neurovirulence and/or neuroinvasivness in JEV. We observed that the E S332G substitution was present in G1A sampled in 2011–2012 along with the NS4B E24G substitution, but neither change was present in the 2018 clade, suggesting these mutations may not be benefical. Further research is needed to fully elucidate the implications of these substitutions for MVEV fitness in vitro and in vivo.

Tiling amplicon viral genome sequencing through multiplexed PCR has been described for orthoflaviviruses including West Nile virus, Yellow Fever virus and Zika virus [40,79,80]. Here we report the first application of this method to MVEV, for a time- and cost-efficient protocol. One of the benefits of this method allows for recovery of genome sequences from bacterially contaminated samples that preclude virus isolation on cell culture. For example, isolate CY2603 was contaminated with bacteria but a full-length CDS could be determined. Isolation of MVEV from clinical samples has been difficult because viremia is short lasting, and most isolates have been derived from postmortem brain tissue [34,60,81] although more recently, MVEV has been isolated from cerebrospinal fluid and urine samples [36]. Application of tiling amplicons to capture low-copy number MVEV in clinical samples could generate more genome data for analysis. In the present study we have applied the tiling amplicon approach to pooled mosquito homogenates and showed that MVEV from PCR-positive, but culture-negative mosquito pools could be sequenced and analyzed as has been done previously for WNV [80].

Complete coding sequences were not obtained for four (K85205, K85508, K91653 & P11645) G2 viruses, due to poor performance of the PCR primer G2 PP6. Binding sites for this primer pair are likely positioned in sub-optimal locations in the 3’ UTR. The poor PCR performance may also be due to limited information regarding G2 sequences at the time of primer design, with only two genomes available for reference [30,33]. Indels in the MVEV UTRs that may influence primer binding have been described [30,82].

In this study we have demonstrated that MVEV is more diverse than previously recognised and exists as four distinct genetic genotypes, with G1 being further divided into three sub-lineages G1A, G1B and the newly described G1C. We confirm MVEV is enzootic in northern WA where transmission has occurred more frequently, and across a wider geographical area, than previously recognised, and we identify regular genotype replacements, possibly linked to genotype-environment interactions, that have occurred over decades where the major genotypes G1 and G2 have circulated in northern Western Australia since the late 1960s. We also show that WA MVEV likely seeded an MVE outbreak in VIC in 1974, further supporting the notion that the enzootic focus of MVEV lies in northern WA. Further research is required to understand MVEV emergence and phylogeography, and how birds spread MVEV within WA and northern Australia, into the east and southeast of the country, and between Australia and PNG and other countries in the Asia-Pacific region.

## Supporting information

S1 Table MVEV sequenced as part of this study. (DOCX)

S2 Table Oligonucleotides used for the generation of MVEV amplicons prior to next-generation sequencing using the Illumina MiSeq platform. (DOCX)

S3 Table Partial MVEV E gene sequences sourced from GenBank for genotype and sub-lineage distribution and abundance assessment. (DOCX)

S4 Table Pairwise nucleotide and amino acid distances between the genotypes and lineages of MVEV for the CDS and all structural and non-structural genes. (DOCX)

S5 Table Results of positive and negative selection pressure analyses of the MVEV CDS dataset using four methods implemented on the DataMonkey.org server. (DOCX)

S1 Fig Map of Australia and Papua New Guinea indicating the geographic origins of MVEV sequences used in this study. Australian states and territories are indicated. The map was created by combining the shapefile of Australia provided by the Australian Bureau of Statistics (available at https://www.abs.gov.au/statistics/standards/australian-statistical-geography-standard-asgs-edition-3/jul2021-jun2026/access-and-downloads/digital-boundary-files/STE_2021_AUST_SHP_GDA2020.zip) with a map of Papua New Guinea (available at https://geojson-maps.kyd.au/) using QGIS v3.14.
(TIFF)

S2 Fig Oligonucleotide binding sites used for the generation of MVEV genotype 1 and 2 amplicons prior to next-generation sequencing with the Illumina MiSeq platform. (TIF)

S3 Fig Maximum likelihood phylogenetic tree of 131 MVEV prM/E gene sequences. Genotypes and subtypes are indicated on the phylogeny, whilst the geographic origins are indicated by colour: New South Wales (NSW), orange; Northern Territory (NT), purple; Queensland (QLD), blue; Victoria (VIC), black; Western Australia (WA), red; Papua New Guinea (PNG), green. The phylogeny was estimated using a general time-reversible model with a gamma distribution (4 categories) and invariant sites. Bootstrap support of nodes is indicated for 1000 replicates, with value ≥ 75% displayed. The phylogeny was rooted using a JEV (GenBank accession number: NC_001437) prM/E gene sequence that was removed to improve visual fidelity.
(TIF)

S4 Fig Maximum likelihood phylogenetic tree of 200 MVEV complete and partial E gene sequences. Genotype and subtypes are indicated on the phylogeny, with those short fragments listed in S3 Table indicated in red. The phylogeny was estimated using a general time-reversible model with a gamma distribution (4 categories) and invariant sites. Bootstrap support of nodes is indicated for 1,000 replicates, with values ≥ 75% displayed.
(TIF)
