# Supplementary material for: Genetic diversity of Murray Valley encephalitis virus 1951–2020 identified via phylogenetic and evolutionary analyses
Source: PLoS Negl Trop Dis. 2025 Jul 3;19(7):e0013181. doi: 10.1371/journal.pntd.0013181 (PMC12240298; doi:10.1371/journal.pntd.0013181)
Supplement: S1 Table — (DOCX) [file pntd.0013181.s001.docx]

Supplemental Table 1: MVEV sequenced as part of this study.

| **Strain** | **Year of isolation** | **Location** | **Species of origin** | **Sequencing method** | **GenBank accession number** |
| --- | --- | --- | --- | --- | --- |
| CY1189 | 1999 | Pormpuraaw, QLD | *Culex sitiens* | TruSeq | OQ418654 |
| CY2603 | 1999 | Pormpuraaw, QLD | *Cx. sitiens* | Nextera | OQ418655 |
| CY2692 | 1999 | Kowanyama, QLD | *Cx. sitiens* | TruSeq | OQ418656 |
| Gu0091 | 2000 | Normanton, QLD | *Cx. annulirostris* | TruSeq | OQ418657 |
| Gu0554 | 2000 | Normanton, QLD | *Cx. annulirostris* | TruSeq | OQ418658 |
| Gu0676 | 2000 | Normanton, QLD | *Cx. annulirostris* | TruSeq | OQ418659 |
| Gu0957 | 2000 | Normanton, QLD | *Cx. annulirostris* | TruSeq | OQ418660 |
| K10341 | 1993 | Willare, Kimberley, WA | *Cx. annulirostris* | Nextera | OQ418661 |
| K10456 | 1993 | Willare, Kimberley, WA | *Cx. annulirostris* | Nextera | OQ418662 |
| K10875 | 1993 | Halls Creek, Kimberley, WA | *Aedes tremulus* | Nextera | OQ418663 |
| K12305 | 1993 | Broome, Kimberley, WA | *Cx. annulirostris* | Nextera | OQ418664 |
| K12309 | 1993 | Broome, Kimberley, WA | *Cx. annulirostris* | Nextera | OQ418665 |
| K12726 | 1993 | Billiluna, Kimberley, WA | *Cx. annulirostris* | Nextera | OQ418666 |
| K12811 | 1993 | Billiluna, Kimberley, WA | *Cx. annulirostris* | Nextera | OQ418667 |
| K13881 | 1993 | Kununurra, Kimberley, WA | *Cx. annulirostris* | Nextera | OQ418668 |
| K14376 | 1993 | Billiluna, Kimberley, WA | *Cx. annulirostris* | Nextera | OQ418669 |
| K14473 | 1993 | Kununurra, Kimberley, WA | *Aedeomyia catasticta* | Nextera | OQ418670 |
| K31302 | 1993 | Wyndham, Kimberley, WA | *Cx. annulirostris* | Nextera | OQ418671 |
| K42307 | 2000 | Billiluna, Kimberley, WA | *Cx. annulirostris* | Nextera | OQ418672 |
| K68196 | 2009 | Fitzroy Crossing, Kimberley, WA | *Cx. annulirostris* | Nextera | OQ418673 |
| K72445 | 2011 | Broome, Kimberley, WA | *Cx. annulirostris* | TruSeq | OQ418674 |
| K72682 | 2011 | Broome, Kimberley, WA | *Cx. annulirostris* | TruSeq | OQ418675 |
| K72974 | 2011 | Broome, Kimberley, WA | *Cx. annulirostris* | TruSeq | OQ418676 |
| K73041 | 2011 | Willare, Kimberley, WA | *Cx. annulirostris* | TruSeq | OQ418677 |
| K73072 | 2011 | Willare, Kimberley, WA | *Cx.* species | TruSeq | OQ418678 |
| K73080 | 2011 | Willare, Kimberley, WA | *Cx. annulirostris* | TruSeq | OQ418679 |
| K73096 | 2011 | Willare, Kimberley, WA | *Cx. annulirostris* | TruSeq | OQ418680 |
| K73146 | 2011 | Derby, Kimberley, WA | *Cx.* species | TruSeq | OQ418681 |
| K73251 | 2011 | Derby, Kimberley, WA | *Cx.* species | TruSeq | OQ418682 |
| K73350 | 2011 | Derby, Kimberley, WA | *Cx. annulirostris* | TruSeq | OQ418683 |
| K73384 | 2011 | Derby, Kimberley, WA | *Cx. annulirostris* | TruSeq | OQ418684 |
| K73475 | 2011 | Derby, Kimberley, WA | *Cx. annulirostris* | TruSeq | OQ418685 |
| K73996 | 2011 | Halls Creek, Kimberley, WA | *Cx.* species | TruSeq | OQ418686 |
| K74361 | 2011 | Kununurra, Kimberley, WA | *Cx. annulirostris* | TruSeq | OQ418687 |
| K74528 | 2011 | Kununurra, Kimberley, WA | *Cx. pullus* | TruSeq | OQ418688 |
| K74558 | 2011 | Kununurra, Kimberley, WA | *Cx. annulirostris* | TruSeq | OQ418689 |
| K74595 | 2011 | Kununurra, Kimberley, WA | *Cx. pullus* | TruSeq | OQ418690 |
| K75564 | 2011 | Central Creek Crossing, Kimberley, WA | *Ae. normanensis* | TruSeq | OQ418691 |
| K75678 | 2011 | Billiluna, Kimberley, WA | *Cx. annulirostris* | TruSeq | OQ418692 |
| K75823 | 2011 | Billiluna, Kimberley, WA | *Cx. annulirostris* | TruSeq | OQ418693 |
| K77160 | 2012 | Fitzroy Crossing, Kimberley, WA | *Cx. annulirostris* | Nextera | OQ418694 |
| K79630 | 2012 | Billiluna, Kimberley, WA | *Cx. annulirostris* | Nextera | OQ418695 |
| K79689 | 2012 | Billiluna, Kimberley, WA | *Cx. annulirostris* | Nextera | OQ418696 |
| K82643 | 2016 | Kununurra, Kimberley, WA | *Cx. annulirostris* | Nextera | OQ418697 |
| K84754 | 2017 | Broome, Kimberley, WA | *Cx. annulirostris* | Nextera | OQ418698 |
| K85133 | 2017 | Billiluna, Kimberley, WA | *Cx. annulirostris* | Nextera | OQ418699 |
| K85205 | 2017 | Billiluna, Kimberley, WA | *Cx. annulirostris* | Nextera | OQ418700 |
| K85508 | 2017 | Billiluna, Kimberley, WA | *Cx. annulirostris* | Nextera | OQ418701 |
| K85511 | 2017 | Billiluna, Kimberley, WA | *Cx. annulirostris* | Nextera | OQ418702 |
| K87401 | 2018 | Broome, Kimberley, WA | *Cx. sitiens* | Nextera | OQ418703 |
| K87857 | 2018 | Broome, Kimberley, WA | *Cx. annulirostris* | Nextera | OQ418704 |
| K89546 | 2018 | Parry’s Creek, Kimberley, WA | *Cx. annulirostris* | Nextera | OQ418705 |
| K90025 | 2018 | Kununurra, Kimberley, WA | *Cx. pullus* | Nextera | OQ418706 |
| K91653 | 2020 | Wyndham, Kimberley, WA | *Cx. annulirostris* | Nextera | OQ418707 |
| K91718 | 2020 | Wyndham, Kimberley, WA | *Cx. annulirostris* | Nextera | OQ418708 |
| OR1 | 1972 | Kununurra, Kimberley, WA | *Cx. annulirostris* | TruSeq | OQ418709 |
| OR2 | 1972 | Kununurra, Kimberley, WA | *Cx. annulirostris* | TruSeq | OQ418710 |
| OR151* | 1973 | Kununurra, Kimberley, WA | *Cx. annulirostris* | TruSeq | OQ418711 |
| OR155 | 1973 | Kununurra, Kimberley, WA | *Cx. annulirostris* | TruSeq | OQ418712 |
| P5140 | 1997 | Newman,  Pilbara, WA | *Cx. annulirostris* | Nextera | OQ418713 |
| P5700 | 1997 | Newman,  Pilbara, WA | *Cx. annulirostris* | TruSeq | OQ418714 |
| P6513 | 2000 | Newman,  Pilbara, WA | *Cx. annulirostris* | Nextera | OQ418715 |
| P6563 | 2000 | Newman,  Pilbara, WA | *Cx.* species | Nextera | OQ418716 |
| P11626 | 2017 | Tom Price,  Pilbara, WA | *Cx. annulirostris* | Nextera | OQ418717 |
| P11645 | 2017 | Tom Price,  Pilbara, WA | *Cx. annulirostris* | Nextera | OQ418718 |
| P11658 | 2017 | Tom Price,  Pilbara, WA | *Cx. annulirostris* | Nextera | OQ418719 |
| P11913 | 2017 | South Hedland, Pilbara, WA | *Cx. annulirostris* | Nextera | OQ418720 |
| PNG6523 | 1998 | Balimo, Western Province, PNG | *Cx. sitiens* group | TruSeq | OQ418721 |
| PNG6910 | 1998 | Balimo, Western Province, PNG | *Cx. sitiens* group | TruSeq | OQ418722 |
| T69 | 1969 | NT | Human | TruSeq | OQ418723 |

*Originally characterized as Koongal-like virus
