# Supplementary material for: Genetic diversity of Murray Valley encephalitis virus 1951–2020 identified via phylogenetic and evolutionary analyses
Source: PLoS Negl Trop Dis. 2025 Jul 3;19(7):e0013181. doi: 10.1371/journal.pntd.0013181 (PMC12240298; doi:10.1371/journal.pntd.0013181)
Supplement: S2 Table — (DOCX) [file pntd.0013181.s002.docx]

Supplemental Table 2: Oligonucleotides used for the generation of MVEV amplicons prior to next-generation sequencing using the Illumina MiSeq platform.

| **Primer Name** | **Sequence (5'-3')** | **Genome position*** | | **Primer Orientation** |
| --- | --- | --- | --- | --- |
|  |  | **Start** | **End** |  |
| MVEV_G1_1_F | CGTGAGCTTCCGATCTCAGT | 14 | 33 | Fwd |
| MVEV_G1_1_R | TGTGTGACTCCCCTGTAGTRGG | 1194 | 1215 | Rev |
| MVEV_G1_2_F | RGGCGATAGTTGCATCACCATC | 1049 | 1070 | Fwd |
| MVEV_G1_2_R | AARACTCCGCCTACTGATCCAA | 2254 | 2275 | Rev |
| MVEV_G1_3_F | AAGCAGATCAATCACCACTGGC | 2142 | 2163 | Fwd |
| MVEV_G1_3_R | GACRGTTGTTCCTGGGCAGTAG | 3308 | 3329 | Rev |
| MVEV_G1_4_F | TGATGCAGTGGAAGAAACGGAG | 3173 | 3194 | Fwd |
| MVEV_G1_4_R | GTGGCTTTTCCCGAGATGACAT | 4339 | 4360 | Rev |
| MVEV_G1_5_F | GAGGTGTTAACAGCAGTGGGAC | 4227 | 4248 | Fwd |
| MVEV_G1_5_R | GACTCTTAGCGGCGACATCART | 5411 | 5432 | Rev |
| MVEV_G1_6_F | GAGGGGCCTTCCAGTTAGGTAT | 5312 | 5333 | Fwd |
| MVEV_G1_6_R | GCATCCGGCCTAACACTTCAAA | 6477 | 6498 | Rev |
| MVEV_G1_7_F | TGAAGCCACGATGGTTAGATGC | 6379 | 6400 | Fwd |
| MVEV_G1_7_R | CGGTACAGAGTATTCCGGCTTC | 7506 | 7527 | Rev |
| MVEV_G1_8_F | AYGCAGTTGTTGATGGCATAGT | 7360 | 7381 | Fwd |
| MVEV_G1_8_R | CGTGGCTGCATACTCTTCCTTY | 8540 | 8561 | Rev |
| MVEV_G1_9_F | CCAGTCAGGTGCTCATTGGAAG | 8389 | 8410 | Fwd |
| MVEV_G1_9_R | GCTTTCAATGTCATCTGGGCCT | 9578 | 9598 | Rev |
| MVEV_G1_10_F | GGAGGAAAGACCGTGATGGATG | 9445 | 9465 | Fwd |
| MVEV_G1_10_R | GATCTGACACGGGCTTCATCTC | 10643 | 10664 | Rev |
| MVEV_G1_11_F | CAGGAGTGGAAACCCTCACAAG | 9771 | 9792 | Fwd |
| MVEV_G1_11_R | GTGCCTTGTGACTGATGTTGGA | 10947 | 10968 | Rev |
| MVEV_G2_1_F | TGATTAACGCGGTTTGAACAGTT | 54 | 76 | Fwd |
| MVEV_G2_1_R | GCCACCGAGGATATTGGGATTT | 1983 | 2004 | Rev |
| MVEV_G2_2_F | AGTGTCGCGTGAAAATGGAGAA | 1833 | 1854 | Fwd |
| MVEV_G2_2_R | ACATCACCCCCGTTGTTTGATT | 3732 | 3753 | Rev |
| MVEV_G2_3_F | ATTGACCCTTTTCAGCTAGGCC | 3548 | 3569 | Fwd |
| MVEV_G2_3_R | TGGTGTCAGGGAATGGATCAGA | 5573 | 5594 | Rev |
| MVEV_G2_4_F | CTGTGCAGAGAGAACATAGCGG | 5346 | 5367 | Fwd |
| MVEV_G2_4_R | AACTGCGTTTTTCATGATGCCG | 7348 | 7369 | Rev |
| MVEV_G2_5_F | GCTTGTTTACGTTGCCCAAAGG | 7152 | 7173 | Fwd |
| MVEV_G2_5_R | GCTCCTAGCCACATGAACCATA | 9111 | 9132 | Rev |
| MVEV_G2_6_F | ATGGAAGAATGCACGAGAAGCC | 8944 | 8965 | Fwd |
| MVEV_G2_6_R | GCCTTGCGACTGATGTTGGAAT | 10884 | 10905 | Rev |

*Genome positions are based on prototype G1 or G2 sequences (GenBank accession No. NC_000943 or KF751871 respectively).
