# Supplementary material for: Genetic diversity of Murray Valley encephalitis virus 1951–2020 identified via phylogenetic and evolutionary analyses
Source: PLoS Negl Trop Dis. 2025 Jul 3;19(7):e0013181. doi: 10.1371/journal.pntd.0013181 (PMC12240298; doi:10.1371/journal.pntd.0013181)
Supplement: S3 Table — (DOCX) [file pntd.0013181.s003.docx]

Supplemental Table 3: Partial MVEV E gene sequences sourced from GenBank for genotype and sub-lineage distribution and abundance assessment.

| **Strain** | **Year of isolation** | **Location** | **Species of origin** | **Genotype** | **Sequence length** | **GenBank accession number** |
| --- | --- | --- | --- | --- | --- | --- |
| GU0054 | 2000 | Normanton, QLD | *Cx. annulirostris* | G1B | 287 | FJ375256 |
| GU0676 | 2000 | Normanton, QLD | *Cx. annulirostris* | G1B | 287 | FJ375257 |
| GU0957 | 2000 | Normanton, QLD | *Cx. annulirostris* | G1B | 287 | FJ375258 |
| 2001326 | 2001 | Mt Isa, QLD | *Cx. annulirostris* | G1B | 287 | FJ375274 |
| 2001356 | 2001 | Mt Isa, QLD | *Cx. annulirostris* | G1B | 287 | FJ375275 |
| BG466 | 1981 | Balgo, WA | *Ae. pseudonormanensis* | G1 | 462 | EF015043 |
| PH260 | 1979 | Newman, WA | *Cx. annulirostris* | G1 | 462 | EF015044 |
| OR655 | 1975 | Kununurra, WA | *Cx. annulirostris* | G1 | 462 | EF015049 |
| CY2603 | 1999 | Pormpuraaw, QLD | *Cx. sitiens* | G1B | 462 | EF015051 |
| P5140 | 1997 | Newman, WA | *Cx. annulirostris* | G1B | 462 | EF015052 |
| K33075 | 1998 | Parry’s Creek, WA | *Cx. annulirostris* | G1B | 462 | EF015055 |
| CY2692 | 1999 | Kowanyama, QLD | *Cx. sitiens* | G1B | 462 | EF015057 |
| K37008 | 1999 | Kununurra, WA | *Cx. pullus* | G1B | 462 | EF015058 |
| P6311 | 1999 | Exmouth, WA | *Cx. annulirostris* | G1B | 462 | EF015059 |
| P7340 | 2002 | Harding Dam, WA | *Cx. annulirostris* | G1B | 462 | EF015061 |
| K4629 | 1990 | Kununurra, WA | *Cx. annulirostris* | G1B | 462 | EF015064 |
| OR314 | 1974 | Kununurra, WA | *Cx. annulirostris* | G1 | 462 | EF015067 |
| K13910 | 1993 | Kununurra, WA | *Cx. annulirostris* | G1C | 462 | EF015068 |
| P9771 | 2009 | Newman, WA | *Cx. annulirostris* | G1A | 675 | JX867136 |
| P9781 | 2009 | WA | N/A | G1A | 675 | JX867137 |
| P9929 | 2009 | WA | N/A | G1A | 675 | JX867138 |
| P9904 | 2009 | Newman, WA | *Cx. annulirostris* | G1A | 675 | JX867139 |
| P9783 | 2009 | Newman, WA | *Cx. annulirostris* | G1A | 675 | JX867140 |
| P9937 | 2009 | Newman, WA | *Cx. annulirostris* | G1A | 675 | JX867141 |
| P9831 | 2009 | WA | N/A | G1A | 675 | JX867142 |
| P9833 | 2009 | WA | N/A | G1A | 675 | JX867143 |
| P9883 | 2009 | WA | N/A | G1A | 675 | JX867144 |
| P9943 | 2009 | Newman, WA | *Cx. annulirostris* | G1A | 675 | JX867145 |
| P9765 | 2009 | Newman, WA | *Cx. annulirostris* | G1A | 675 | JX867146 |
| P9978 | 2009 | Newman, WA | *Cx.* species | G1A | 675 | JX867147 |
| P9808 | 2009 | Newman, WA | *Cx.* species | G1A | 675 | JX867148 |
| P9950 | 2009 | Newman, WA | *Cx.* species | G1A | 675 | JX867149 |
| P9705 | 2009 | Port Hedland, WA | *Cx. annulirostris* | G1A | 675 | JX867150 |
| P9946 | 2009 | WA | N/A | G1A | 675 | JX867151 |
| P9753 | 2009 | WA | N/A | G1A | 675 | JX867152 |
| P9652 | 2009 | Port Hedland, WA | *Cx. annulirostris* | G1A | 675 | JX867153 |
| P9777 | 2009 | Newman, WA | *Cx.* species | G1B | 675 | JX867155 |
| P9986 | 2009 | Newman, WA | *Cx. annulirostris* | G1B | 675 | JX867156 |
| P9754 | 2009 | WA | N/A | G1B | 675 | JX867157 |
| P9901 | 2009 | Newman, WA | *Cx.* species | G1B | 675 | JX867158 |
| P9749 | 2009 | WA | N/A | G1B | 675 | JX867159 |
| P9992 | 2009 | Newman, WA | *Cx. annulirostris* | G1B | 675 | JX867161 |
| K68834 | 2009 | Kununurra, WA | *Cx. annulirostris* | G1A | 675 | JX867162 |
| K69679 | 2009 | Kununurra, WA | *Cx. annulirostris* | G1A | 675 | JX867163 |
| K69211 | 2009 | Kununurra, WA | *Cx. annulirostris* | G1A | 675 | JX867164 |
| K67259 | 2009 | Wyndham, WA | *Cx. annulirostris* | G1A | 675 | JX867166 |
| K67317 | 2009 | Wyndham, WA | *Cx. annulirostris* | G1A | 675 | JX867167 |
| K67238 | 2009 | Carnarvon, WA | *Cx. annulirostris* | G1A | 675 | JX867168 |
| K68439 | 2009 | Wyndham, WA | *Cx. annulirostris* | G1A | 675 | JX867170 |
| K69016 | 2009 | Kununurra, WA | *Cx. annulirostris* | G1A | 675 | JX867172 |
| K68260 | 2009 | Halls Creek, WA | *Cx. annulirostris* | G1A | 675 | JX867173 |
| K68463 | 2009 | Wyndham, WA | *Cx. annulirostris* | G1A | 675 | JX867175 |
| K68474 | 2009 | WA | N/A | G1A | 675 | JX867177 |
| K68970 | 2009 | Kununurra, WA | *Cx. annulirostris* | G1A | 675 | JX867178 |
| K69155 | 2009 | Kununurra, WA | *Cx. pullus* | G1A | 675 | JX867180 |
| K69051 | 2009 | Kununurra, WA | *Cx. pullus* | G1A | 675 | JX867181 |
| K69052 | 2009 | Kununurra, WA | *Cx. pullus* | G1A | 675 | JX867182 |
| K69521 | 2009 | Kununurra, WA | *Cx. pullus* | G1A | 675 | JX867183 |
| K69381 | 2009 | Kununurra, WA | *Cx. pullus* | G1A | 675 | JX867184 |
| K69612 | 2009 | Kununurra, WA | *Cx. pullus* | G1A | 675 | JX867185 |
| K66298 | 2008 | Kununurra, WA | *Cx. pullus* | G1A | 675 | JX867187 |
| K67234 | 2008 | Carnarvon, WA | *Cx. annulirostris* | G1A | 675 | JX867188 |
| K67235 | 2008 | Carnarvon, WA | *Cx. annulirostris* | G1A | 675 | JX867189 |
| K61375 | 2006 | Derby, WA | *Cx. annulirostris* | G1B | 675 | JX867191 |
| K60555 | 2006 | Kununurra, WA | *Cx. annulirostris* | G1B | 675 | JX867192 |
| K59582 | 2006 | Fitzroy Crossing, WA | *Cx. annulirostris* | G1B | 675 | JX867193 |
| K61299 | 2006 | Kununurra, WA | *Cx. annulirostris* | G1B | 675 | JX867194 |
| K60748 | 2006 | Wyndham, WA | *Cx. annulirostris* | G1B | 675 | JX867195 |
| K61396 | 2006 | Derby, WA | *Cx. annulirostris* | G1B | 675 | JX867196 |
| P8891 | 2006 | Karratha, WA | *Ae. normanensis* | G1B | 675 | JX867199 |
| P8400 | 2006 | Newman, WA | *An. amictus* | G1B | 675 | JX867200 |
| 145432 | 2008 | Griffith, NSW | *Cx. annulirostris* | G1B | 680 | FJ037723 |
| 145505 | 2008 | Griffith, NSW | *Cx. annulirostris* | G1B | 689 | FJ037718 |
| 145628 | 2008 | Griffith, NSW | *Cx. annulirostris* | G1B | 706 | FJ037715 |

N/A – Not Available, NSW – New South Wales, WA – Western Australia, QLD – Queensland, *Ae.* – *Aedes*, *An.* – *Anopheles*, *Cx.* – *Culex*.
