# Supplementary material for: Genetic diversity of Murray Valley encephalitis virus 1951–2020 identified via phylogenetic and evolutionary analyses
Source: PLoS Negl Trop Dis. 2025 Jul 3;19(7):e0013181. doi: 10.1371/journal.pntd.0013181 (PMC12240298; doi:10.1371/journal.pntd.0013181)
Supplement: S4 Table — (DOCX) [file pntd.0013181.s004.docx]

| Genotype/  Lineage | % Range of nucleotide (amino acid) identities | | | | | | | | | | |
| --- | --- | --- | --- | --- | --- | --- | --- | --- | --- | --- | --- |
|  | CDS | Capsid | prM | Envelope | NS1 | NS2A | NS2B | NS3 | NS4A | NS4B | NS5 |
| Intra genotype | | | | | | | | | | | |
| G1 | 94.9 (98.4) - 100 | 94.9 (94.4) - 100 | 95.2 (98.2) - 100 | 93.9 (98.6) - 100 | 95.3 (98.0) - 100 | 96.9 (99.1) - 100 | 91.9 (95.4) - 100 | 94.5 (98.2) - 100 | 96.0 (99.3) - 100 | 93.3 (96.1) - 100 | 95.7 (98.6) - 100 |
| 1A | 97.5 (99.2) - 100 | 98.1 (97.6) - 100 | 97.6 (98.8) - 100 | 96.9 (99.2) - 100 | 97.8 (99.4) - 100 | 97.4 (99.1) - 100 | 96.2 (99.2) - 100 | 97.4 (98.9) - 100 | 96.6 (99.3) - 100 | 96.8 (96.7) - 100 | 97.5 (98.9) - 100 |
| 1B | 98.3 (99.3) - 99.9 (99.9) | 97.6 (98.4) - 100 | 98.4 (99.4) - 100 | 98.0 (99.2) - 100 | 98.0 (99.1) - 99.9 (100) | 98.4 (98.2) - 99.9 (100) | 96.7 (97.7) - 100 | 98.0 (98.9) - 100 | 98.0 (100) - 100 | 97.3 (98.1) - 100 | 98.7 (99.4) - 100 |
| 1C | 99.5 (99.7) - 100 | 98.9 (98.4) - 100 | 99.4 (98.8) - 100 | 99.5 (99.4) - 100 | 99.8 (99.4) - 100 | 99.6 (100) - 100 | 99.0 (99.2) - 100 | 99.2 (99.7) - 100 | 99.6 (100) - 100 | 99.1 (99.2) - 100 | 99.6 (99.8) - 100 |
| G2 | 96.7 (99.0) - 100 | 96.8 (98.4) - 100 | 97.2 (100) - 100 | 95.9 (99.2) - 100 | 96.9 (98.9) - 100 | 95.6 (98.2) - 100 | 95.9 (96.2) - 100 | 97.1 (99.4) - 99.9 (100) | 96.6 (97.3) - 100 | 95.9 (97.3) - 100 | 96.7 (99.6) - 100 |
| Between 1A & 1B | 94.9 (98.4) - 96.1 (99.1) | 94.9 (94.4) - 97.3 (96.8) | 95.2 (98.2) - 97.0 (99.4) | 93.9 (98.6) - 95.5 (99.4) | 95.3 (98.0) - 97.3 (99.1) | 95.4 (98.2) - 97.1 (99.6) | 91.9 (95.4) - 95.7 (98.5) | 94.5 (98.2) - 96.1 (99.4) | 94.0 (99.3) - 96.4 (100) | 93.3 (96.1) - 95.6 (98.8) | 95.7 (98.6) - 96.5 (99.4) |
| Between 1A & 1C | 96.2 (98.8) - 96.8 (99.4) | 96.0 (96.8) - 97.6 (98.4) | 96.8 (98.8) - 98.4 (100) | 95.5 (98.6) - 96.6 (99.4) | 96.4 (98.6) - 97.2 (99.1) | 96.9 (99.6) - 97.7 (100) | 93.9 (97.7) - 96.4 (99.2) | 95.9 (98.5) - 96.6 (99.7) | 96.0 (98.7) - 97.5 (99.3) | 94.3 (96.9) - 95.6 (98.8) | 96.7 (99.0) - 97.2 (99.8) |
| Between 1B & 1C | 96.3 (98.8) - 97.1 (99.2) | 95.7 (95.2) - 97.6 (98.4) | 96.2 (98.2) - 97.0 (99.4) | 95.9 (98.8) - 96.9 (99.6) | 95.7 (98.9) - 97.2 (100) | 97.1 (98.7) - 98.1 (99.6) | 94.4 (96.2) - 96.7 (99.2) | 96.1 (98.5) - 97.1 (99.7) | 96.0 (99.3) - 97.8 (99.3) | 94.1 (96.9) - 95.9 (98.1) | 96.9 (99.0) - 97.5 (99.4) |
| Inter genotype | | | | | | | | | | | |
| Between G1 & G2 | 86.2 (95.7) - 87.7 (96.7) | 87.5 (90.4) - 91.2 (93.6) | 86.0 (98.8) - 89.4 (100) | 84.7 (94.2) - 87.0 (95.6) | 86.9 (96.6) - 89.2 (97.7) | 84.3 (96.0) - 87.7 (97.4) | 83.5 (90.8) - 89.3 (96.9) | 85.8 (95.8) - 87.9 (97.3) | 85.9 (93.3) - 88.1 (96.6) | 82.9 (90.7) - 84.9 (94.6) | 87.2 (96.9) - 88.9 (98.0) |
| Between G1 & G3 | 89.1 (97.6) - 90.3 (98.4) | 89.6 (90.4) - 91.7 (92.8) | 89.6 (98.2) - 91.8 (99.4) | 88.6 (97.4) - 90.2 (98.2) | 88.0 (96.9) - 89.2 (98.0) | 90.0 (99.1) - 90.7 (99.6) | 87.3 (96.2) - 89.1 (99.2) | 88.6 (97.7) - 89.9 (98.9) | 89.0 (98.0) - 90.4 (98.7) | 86.4 (96.1) - 88.4 (98.1) | 89.9 (98.0) - 91.6 (98.8) |
| Between G1 & G4 | 89.6 (97.9) - 91.1 (98.6) | 89.3 (93.6) - 91.5 (95.2) | 89.0 (98.8) - 90.8 (100) | 88.5 (97.8) - 90.6 (98.6) | 90.2 (98.6) - 91.8 (99.7) | 89.1 (98.7) - 90.6 (99.1) | 87.5 (96.2) - 89.8 (99.2) | 89.5 (97.7) - 90.7 (98.9) | 89.3 (98.7) - 90.4 (99.3) | 87.1 (95.0) - 89.8 (97.3) | 90.2 (98.0) -92.1 (98.7) |
| Between G2 & G3 | 86.7 (96.0) - 87.6 (96.5) | 87.7 (88.8) - 90.4 (89.6) | 87.0 (99.4) - 88.2 (99.4) | 86.1 (95.0) - 87.7 (95.8) | 86.6 (96.0) - 87.3 (96.3) | 86.9 (96.9) - 88.4 (97.8) | 83.7 (93.9) - 86.3 (96.2) | 86.8 (96.9) - 87.2 (97.4) | 86.4 (92.6) - 87.9 (95.3) | 83.1 (92.6) - 85.0 (95.0) | 88.0 (97.3) - 88.4 (97.6) |
| Between G2 & G4 | 87.0 (96.3) - 87.9 (96.7) | 87.2 (90.4) - 89.9 (91.2) | 86.4 (100) - 88.0 (100) | 86.6 (95.4) - 88.0 (95.8) | 87.6 (98.0) - 88.3 (97.7) | 88.1 (97.4) - 89.6 (97.8) | 84.0 (93.9) - 87.3 (96.2) | 86.6 (96.9) - 87.5 (97.4) | 86.6 (93.3) - 87.7 (96.0) | 83.7 (92.2) - 84.5 (93.8) | 88.0 (97.1) - 88.7 (97.3) |
| Between G3 & G4 | 93.7 (99.0) | 94.9 (92.8) | 94.0 (99.4) | 93.0 (99.2) | 92.2 (98.3) | 94.4 (99.6) | 93.4 (100) | 93.9 (100) | 94.0 (98.0) | 92.5 (97.7) | 94.5 (99.4) |

Supplemental Table 4: Pairwise nucleotide and amino acid distances between the genotypes and lineages of MVEV for the CDS and all structural and non-structural genes.
