# Supplementary material for: Genetic diversity of Murray Valley encephalitis virus 1951–2020 identified via phylogenetic and evolutionary analyses
Source: PLoS Negl Trop Dis. 2025 Jul 3;19(7):e0013181. doi: 10.1371/journal.pntd.0013181 (PMC12240298; doi:10.1371/journal.pntd.0013181)
Supplement: S5 Table — (DOCX) [file pntd.0013181.s005.docx]

Supplemental Table 5: Results of positive and negative selection pressure analyses of the MVEV CDS dataset using four methods implemented on DataMonkey.org server.

| Method | Selection | |
| --- | --- | --- |
|  | Positive (diversifying) | Negative |
| SLAC^a^ | 0 | 257 |
| FEL^a^ | 1 | 883 |
| FUBAR^b^ | 2 | 2713 |
| MEME^c^ | 3 | NA |

^a^Number of sites where *P* < 0.05

^b^Number of sites where posterior probability (β > α) > 0.9

^c^Number of sites where *P* <0.01

NA = Not Applicable
