# Supplementary figures and images for: Genetic diversity of Murray Valley encephalitis virus 1951–2020 identified via phylogenetic and evolutionary analyses
Source: PLoS Negl Trop Dis. 2025 Jul 3;19(7):e0013181. doi: 10.1371/journal.pntd.0013181 (PMC12240298; doi:10.1371/journal.pntd.0013181)

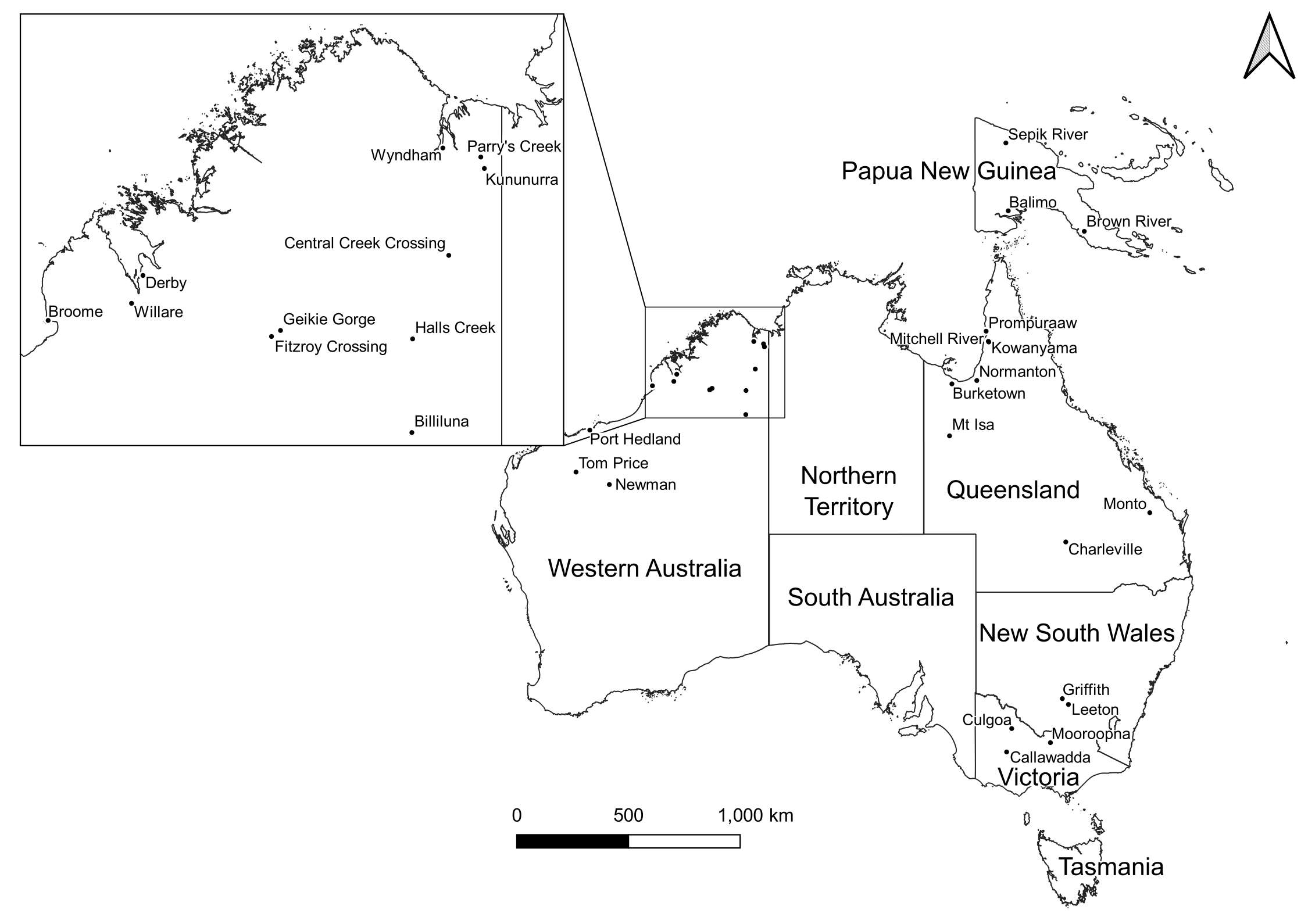

Supplement: S1 Fig — Australian states and territories are indicated. The map was created by combining the shapefile of Australia provided by the Australian Bureau of Statistics (available at https://www.abs.gov.au/statistics/standards/australian-statistical-geography-standard-asgs-edition-3/jul2021-jun2026/access-and-downloads/digital-boundary-files/STE_2021_AUST_SHP_GDA2020.zip) with a map of Papua New Guinea (available at https://geojson-maps.kyd.au/) using QGIS v3.14. (TIFF) [file pntd.0013181.s006.tiff]

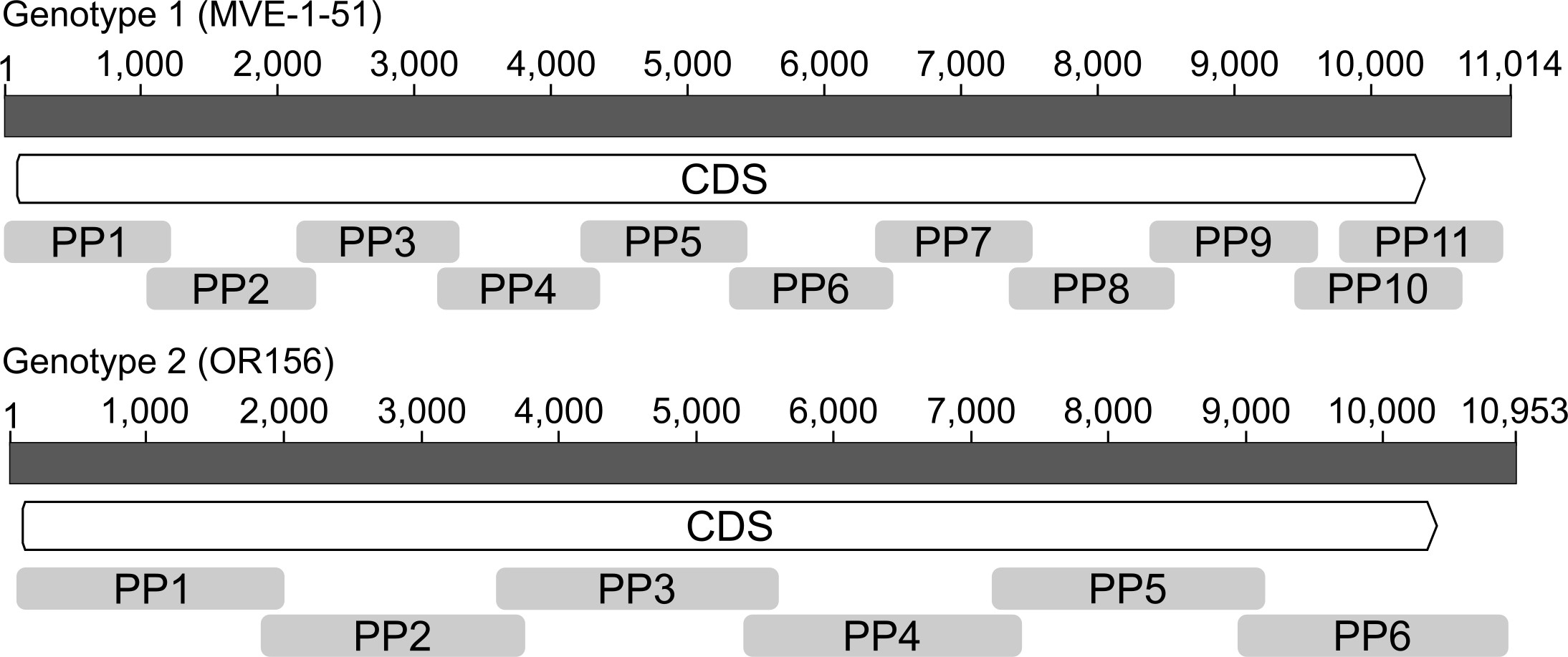

Supplement: S2 Fig — (TIF) [file pntd.0013181.s007.tif]

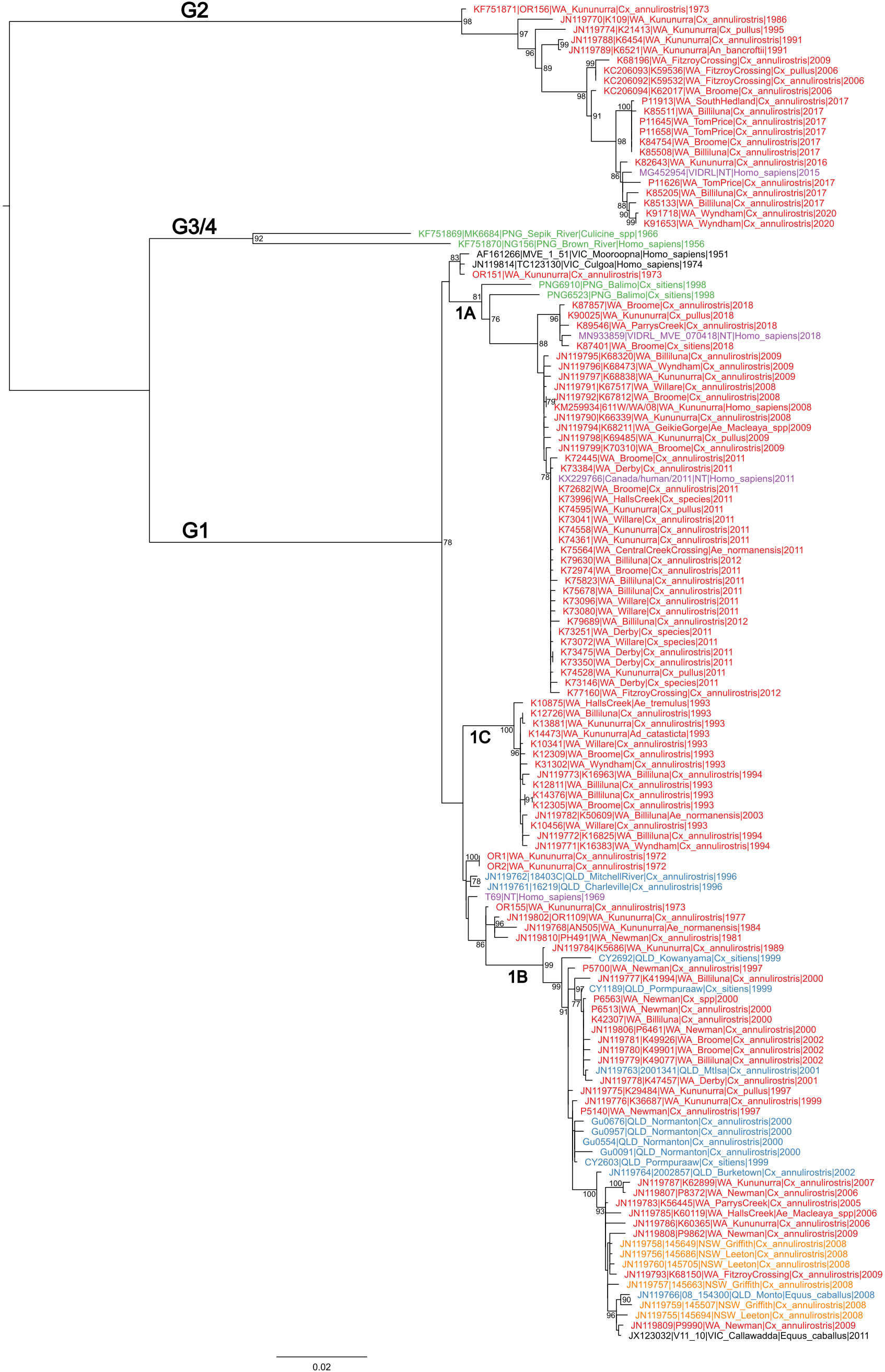

Supplement: S3 Fig — Genotypes and subtypes are indicated on the phylogeny, whilst the geographic origins are indicated by colour: New South Wales (NSW), orange; Northern Territory (NT), purple; Queensland (QLD), blue; Victoria (VIC), black; Western Australia (WA), red; Papua New Guinea (PNG), green. The phylogeny was estimated using a general time-reversible model with a gamma distribution (4 categories) and invariant sites. Bootstrap support of nodes is indicated for 1000 replicates, with value ≥ 75% displayed. The phylogeny was rooted using a JEV (GenBank accession number: NC_001437) prM/E gene sequence that was removed to improve visual fidelity. (TIF) [file pntd.0013181.s008.tif]

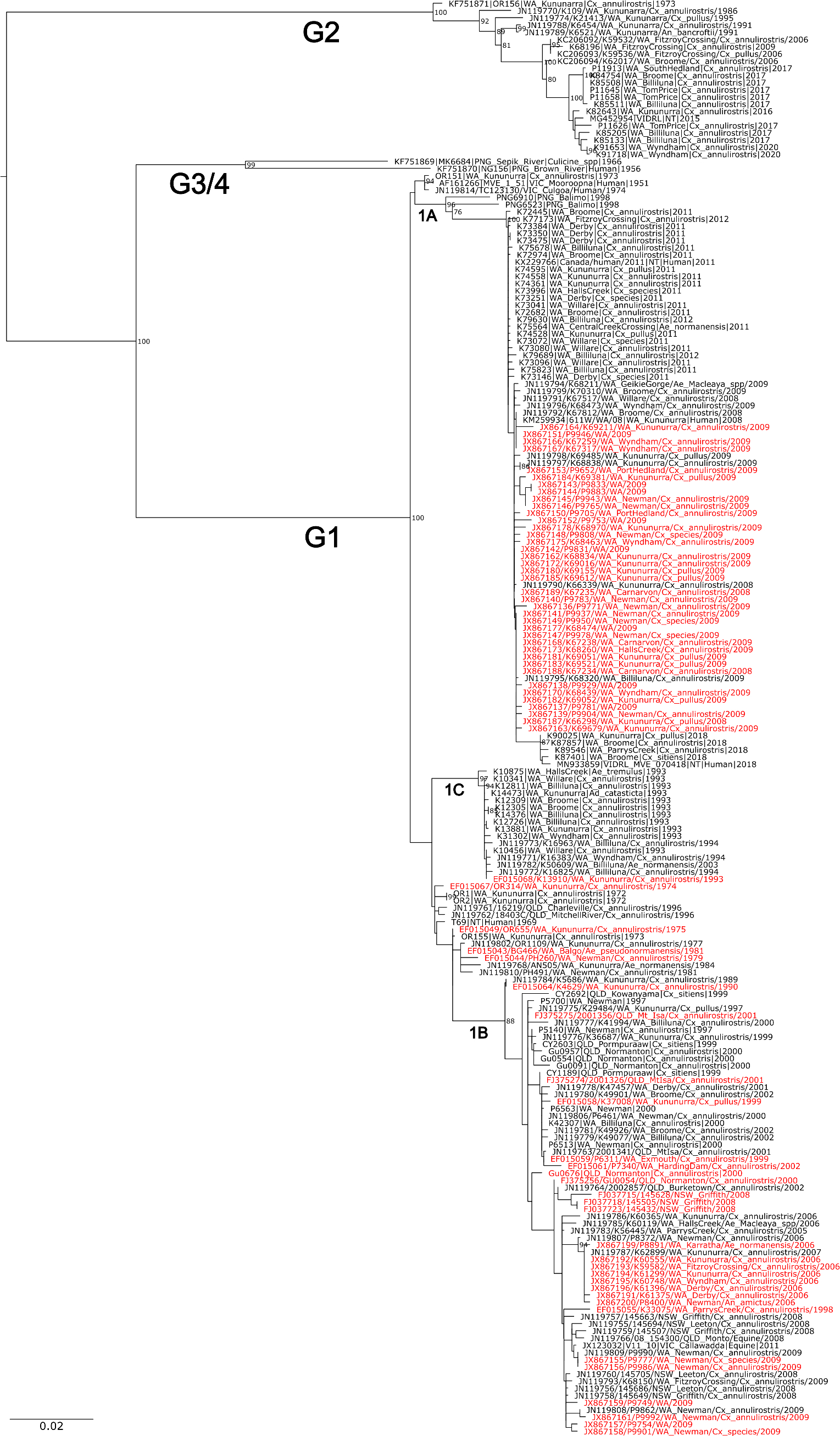

Supplement: S4 Fig — Genotype and subtypes are indicated on the phylogeny, with those short fragments listed in S3 Table indicated in red. The phylogeny was estimated using a general time-reversible model with a gamma distribution (4 categories) and invariant sites. Bootstrap support of nodes is indicated for 1,000 replicates, with values ≥ 75% displayed. (TIF) [file pntd.0013181.s009.tif]
